# Case report: Severe respiratory failure caused by licorice

**DOI:** 10.3389/fphar.2023.1289755

**Published:** 2023-11-23

**Authors:** Hiroaki Taniguchi, Takero Terayama, Nobuaki Kiriu, Hiroshi Kato, Soichiro Seno, Yasumasa Sekine, Yoshihiro Tanaka, Tetsuro Kiyozumi

**Affiliations:** ^1^ Department of Traumatology and Critical Care Medicine, National Defense Medical College, Tokorozawa, Japan; ^2^ Department of Emergency Self-Defense Forces Central Hospital, Tokyo, Japan

**Keywords:** licorice, herbal medicine, pseudoaldosteronism, apparent mineralocorticoid excess, diaphragm, hypercapnia

## Abstract

Licorice, one of the most commonly used herbs, can cause hypokalemia, metabolic alkalosis, and apparent mineralocorticoid excess, also known as pseudoaldosteronism. Herein, we present a case of diaphragmatic dysfunction caused by licorice administration. An 80-year-old woman who had been taking dietary supplements and following a restricted diet for approximately 6 months was brought to the emergency department with impaired consciousness. Chronic respiratory acidosis was observed, and hypertension and hypokalemia became more prominent during hospitalization. History revealed that she was taking herbal medicines containing licorice. Based on the results of hormone tests, the patient was diagnosed with pseudoaldosteronism. Chest radiography and pulmonary function tests confirmed the clinical diagnosis of diaphragmatic dysfunction. The metabolic alkalosis resulting from licorice administration may have contributed to the impairment of the respiratory muscles. This case suggests that caution should be exercised when using licorice in patients with preexisting health or medical issues such as advanced age, malnutrition, and electrolyte imbalance.

## 1 Introduction

Licorice, one of the oldest and most commonly used herbs, has been extensively studied for its potential benefits in promoting recovery and protection of various body systems, including the nervous, digestive, respiratory, endocrine, and cardiovascular systems (Yang et al., 2015). However, licorice can have side effects, including excessive mineralocorticoid activity, which can manifest as sodium retention, hypertension, hypokalemia, metabolic alkalosis, and decreased serum renin and aldosterone activity (Stewart, 2003). The association between licorice and respiratory failure remains relatively unexplored, although several case reports have described this relation (Yasue et al., 2007; Shibata et al., 2022).

Herein, we present a case highlighting the serious adverse effects of licorice administration, which resulted in diaphragmatic dysfunction.

## 2 Case description

An 80-year-old woman had a history of bronchial asthma that was managed well without medication. She experienced constipation and mobility difficulties a few days before admission but did not seek medical attention. Her condition eventually deteriorated, and she became unconscious and was brought to the emergency department with shallow breathing.

Upon arrival, her consciousness level was Glasgow Coma Scale 4 (E2V1M1). Her blood pressure was 164/90 mmHg, heart rate was 117/min, and respiratory rate was 20/min with shallow breathing. The oxygen saturation level was 96%, and the patient was receiving oxygen at 10 L/min through a reservoir mask. Arterial blood gas analysis and blood tests were also performed (Table 1), which indicated a pH of 7.245, PaCO_2_ of 97.1 mmHg, PaO_2_ of 52.9 mmHg, and HCO_3_^−^ concentration of 42.5 mmol/L. These findings were suggestive of chronic respiratory acidosis with renal compensation. The blood samples revealed hypokalemia despite acidosis and high c-reactive protein. Calcium measured at 9.2 mg/dL was within the normal range; magnesium levels were not assessed.

**TABLE 1:** Blood test results on admission.

Variables	Results
Arterial blood gas analysis^a^	
pH	7.245
PaCO_2_ (mmHg)	97.1
PaO_2_ (mmHg)	52.9
HCO_3_ (mmol/L)	42.5
Base excess (mmol/L)	14.9
Lactate (mmol/L)	2.7
Complete blood cell count	
White blood cell count (×10^3^/μL)	9.1
Hemoglobin (g/dL)	12.7
Platelet count (×10^4^/μL)	26.4
Blood biochemistry	
Total bilirubin (mg/dL)	0.49
AST (U/L)	31
ALT (U/L)	26
Creatinine (mg/dL)	0.75
Sodium (mmol/L)	144
Potassium (mmol/L)	3.2
Chloride (mmol/L)	95
Calcium (mg/dL)	9.2
C-reactive protein (mg/dL)	12.1
Blood glucose (mg/dL)	178
TSH(μIU/mL)	4.56
free T3 (pg/mL)	1.88
free T4 (pg/mL)	1.20

AST, aspartate aminotransferase; ALT, alanine aminotransferase.

^a^ Blood gas analysis was performed while administering oxygen at a rate of 10 L/min via a mask with a reservoir.

**FIGURE 1:**
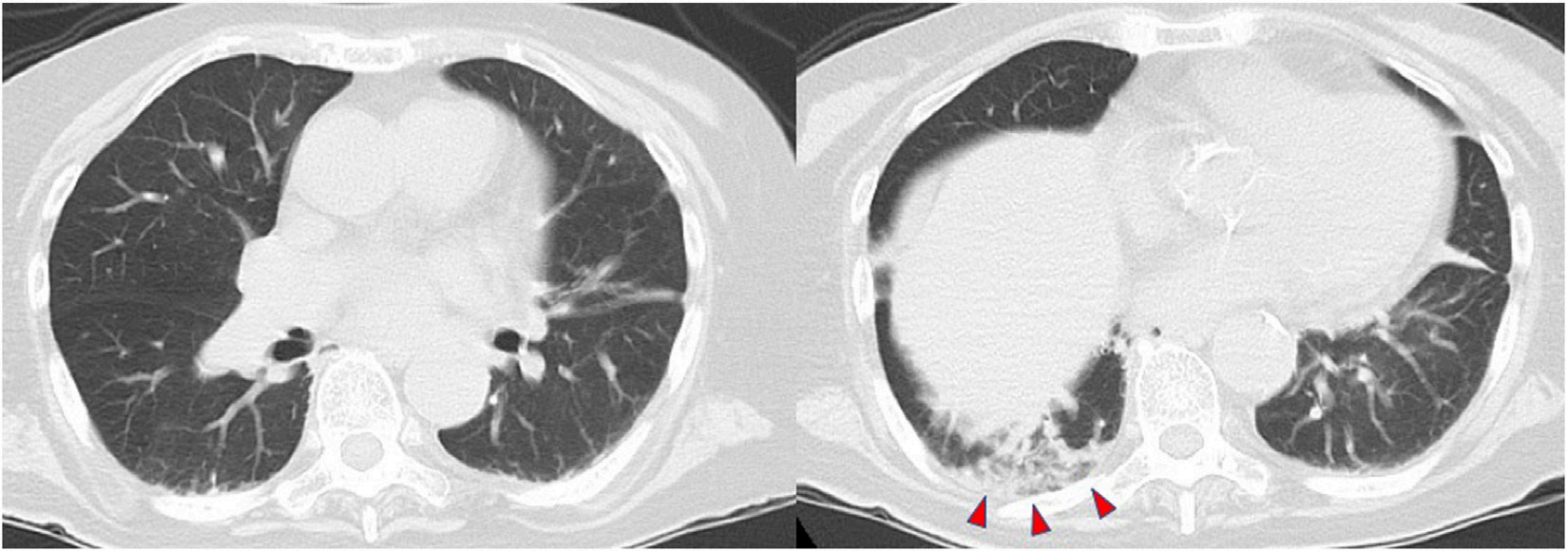
Computed tomography of the chest shows slight consolidation at the base of the right lung (red arrowheads).

Computed tomography (CT) of the chest demonstrated slight consolidation at the base of the right lung, with no evidence of severe emphysema, pneumonia, or atelectasis (Figure 1). In addition, there were no organic abnormalities in the brain, cervical spinal cord, or endocrine organs such as the thyroid and adrenal glands. Magnetic resonance imaging (MRI) also showed no abnormal signals in the brain and cervical spinal cord. Cerebrospinal fluid examination ruled out a central nervous system infection. She was diagnosed with a left ureteral stone and an *Escherichia coli* urinary tract infection, which led to bacteremia. Based on these findings, the patient was placed on a ventilator, and administered ceftriaxone. After endotracheal intubation, the PaO_2_ quickly increased to 481.1 mmHg under the fraction of inspired oxygen 1.0, suggesting severe hypoventilation rather than shunt disease as the cause of hypoxemia.

On the second day of admission, hypertension and hypokalemia became more pronounced, necessitating antihypertensive medications and potassium supplementation. On the third day, the patient was extubated; however, hypercapnia persisted. Initially, noninvasive ventilation was employed to address the respiratory distress. However, for comfort and acceptability, a high-flow nasal cannula that delivered oxygen at a fraction of 0.3 with a flow rate of 30 L/min was used to maintain adequate oxygenation. An interview revealed that she had been taking herbal medicine containing 0.5 g/day of licorice for analgesia for the past 6 months. Moreover, she had been taking dietary supplements and following a restricted diet in an attempt to lose weight for approximately 6 months before admission. The extent of weight loss is unknown, but at admission, she was 150 cm tall and weighed 40 kg. Hormone measurements conducted in the early morning while in a supine position showed normal lower limits for plasma renin activity (0.4 ng/mL/h; range, 0.2–2.3) and aldosterone (5.2 pg/mL; range, 4.0–82.1), leading to a diagnosis of pseudoaldosteronism. Mild hypothyroidism was noted (TSH 4.56 μIU/mL; range, 0.61–4.23; free T3 level 1.88 pg/mL; range, 2.51–3.47; free T4 level 1.20 pg/mL; range, 0.68–1.26), although the association with respiratory acidosis remained unclear.

**FIGURE 2:**
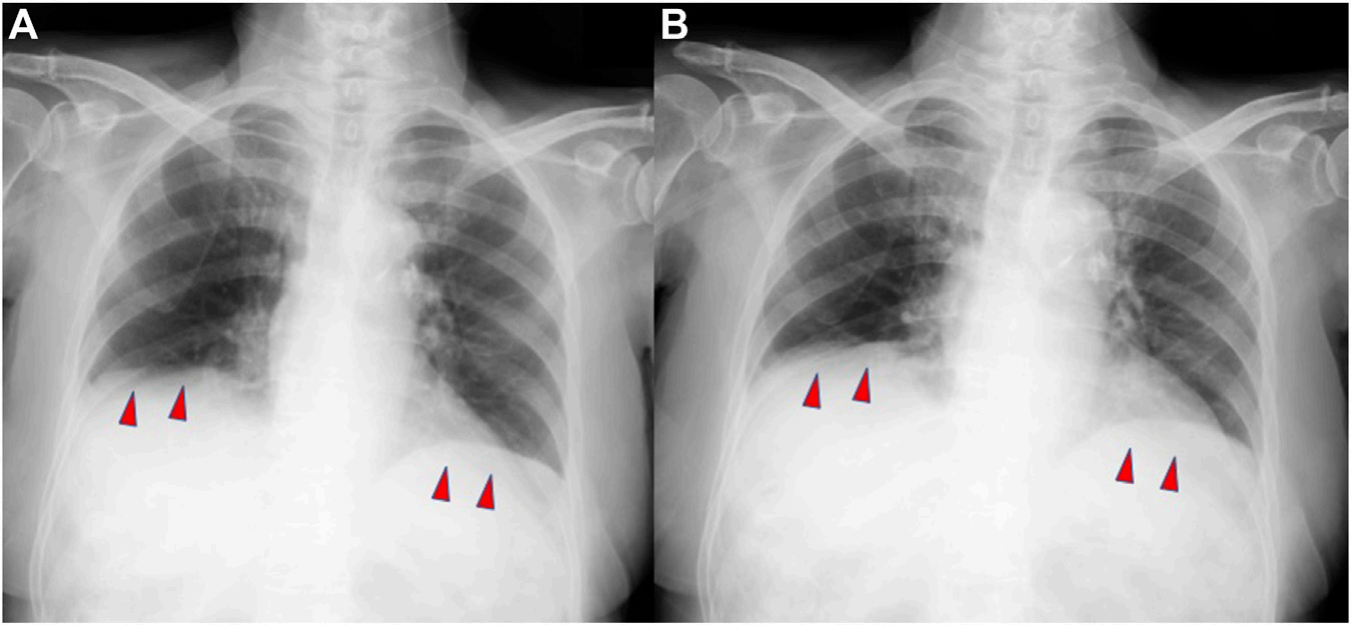
Plain chest radiographs demonstrating minimal diaphragmatic movement during maximum inspiration **(A)** and maximum expiration **(B)**.

Respiratory function evaluation on the ninth day of admission (after 6 days of extubation) revealed combined ventilatory impairment, with forced vital capacity (FVC) of 1.05 L, predicted forced vital capacity (%FVC) of 50.9%, and forced expiratory volume in 1 s (FEV1%_(G)_) of 50.3% (Table 2). Furthermore, physical examination revealed paradoxical abdominal movements and breathing. Chest radiography during the maximum inspiration and expiration revealed no diaphragmatic movement, suggesting that severe diaphragmatic dysfunction still occurred after extubation (Figure 2). On the 16th day of hospitalization, the patient was transferred to another facility for respiratory rehabilitation, because no additional treatment was required after extubation except for licorice cessation. Eleven days after the transfer, she passed away although the cause of death remains unknown.

**TABLE 2:** Pulmonary function test results on the ninth day of admission.

Respiratory parameter	Measurement	Unit
FEV1	0.66	L/s
FEV1%(G)	63.4	%
FVC	1.05	L
%FVC	50.3	%
VC	1.05	L
%VC	50.9	%

FEV1, forced expiratory volume in 1 s; FVC, forced vital capacity; VC, vital capacity.

FEV1%(G), predicted forced expiratory volume in 1 s (Gaensler’s method; calculated using the formula FEV1/FVC × 100).

## 3 Discussion

This case highlights the possibility of licorice-induced pseudoaldosteronism, metabolic alkalosis, and hypokalemia leading to diaphragmatic dysfunction. Notably, in specific patient populations, such as those with prolonged gastrointestinal transit time, advanced age, and female sex, the administration of licorice can unexpectedly result in respiratory muscle impairment due to drug-induced metabolic alkalosis and hypokalemia.

Licorice, a medicinal herb containing more than 20 triterpenoids and 300 flavonoids, is widely used in Chinese medicine owing to its various beneficial properties, including antitumor, antibacterial, antiviral, anti-inflammatory, and immunomodulatory effects (Yang et al., 2015). It also has known side effects, including sodium retention resulting from mineralocorticoid excess, hypertension, hypokalemia, and metabolic alkalosis (Farese et al., 1991). Licorice undergoes hydrolysis in the intestinal tract and is primarily absorbed as glycyrrhetinic acid (GRA). The half-life of GRA ranges from 10 h to 30 h (Krähenbühl et al., 1994). However, when 130 mg/day of GRA was repeated for 5 days, it took 4 days after cessation for the urinary cortisol/cortisone ratio to normalize (Ploeger et al., 2001). This suggests that prolonged internal use of licorice may lead to extended effects after discontinuation.

Consumption of licorice leads to the inhibition of the 11-beta-hydroxysteroid dehydrogenase enzyme type 2 isoform, which suppresses the conversion of cortisol to cortisone at aldosterone-binding sites, such as the renal cortical collecting ducts. As a result, cortisol is not metabolized, causing functional mineralocorticoid excess (Stewart, 2003). The incidence of this side effect may be related to the dose and duration of licorice administration (Homma et al., 2006), and factors such as hypokalemia, prolonged gastrointestinal transit time, reduced type 2 11-β-hydroxysteroid dehydrogenase activity, hypertension, neuroleptic anorexia, advanced age, and female sex (Nazari et al., 2017). The specific doses of licorice and GRA that cause pseudoaldosteronism are unknown. However, it has been reported that blood pressure is elevated even at doses that are generally considered low, but at higher concentrations than what is described in this case. (Sigurjonsdottir et al., 2001).

The diaphragm, innervated by the phrenic nerve, is the primary respiratory muscle. Dysfunction of this muscle can lead to respiratory failure, and common causes include neuromuscular disorders and structural abnormalities (Laghi and Tobin, 2003). In addition, metabolic abnormalities such as malnutrition, hypophosphatemia, hypomagnesemia, hypokalemia, hypocalcemia, and thyroid disorders have been identified as potential causes of diaphragmatic dysfunction (McCool and Tzelepis, 2012). In critically ill patients with sepsis and multiple organ failure, other differential diagnoses for diaphragmatic dysfunction include polyneuritis and myopathy. These conditions should be considered in patients experiencing difficulty weaning from respiratory support (Chawla and Gruener, 2010). Chest radiographs are commonly used for diagnosis and are considered to have high sensitivity but low specificity (Chetta et al., 2005). Severe diaphragmatic weakness or bilateral diaphragmatic paralysis can typically be observed as a reduction of 30%–50% of the predicted total lung volume on pulmonary function tests (McCool and Tzelepis, 2012).

In this case, all physiological evaluations, including pulmonary function tests, chest radiographs, and analysis of breathing patterns, suggested diaphragmatic dysfunction. It is plausible that hypokalemia and metabolic alkalosis due to licorice-induced pseudoaldosteronism could have played a role in precipitating diaphragmatic dysfunction. Initially, the respiratory acidosis was suspected to be caused by severe hypoventilation due to excessive respiratory compensation in response to metabolic alkalosis associated with pseudoaldosteronism. However, a more meticulous evaluation was necessary because conclusive evidence attributing it solely to heightened respiratory compensation was lacking. The hypothesis that licorice-induced pseudoaldosteronism could underlie diaphragmatic dysfunction holds merit. Although previous reports have suggested a potential link between licorice and respiratory failure (Yasue et al., 2007; Shibata et al., 2022), the lack of a specific report of diaphragmatic dysfunction makes the hypothesis novel. Neither central nervous system abnormality nor pulmonary disease was discovered to elucidate the type 2 respiratory failure of the patient, reinforcing our hypothesis. Despite the minimal intake of licorice, factors such as advanced age, restricted diet for 6 months, augmented pseudoaldosteronism due to constipation, and worsened general condition due to sepsis must be involved in the mechanisms of her diaphragmatic dysfunction.

This case report has some limitations. First, we were unable to definitively exclude all potential causes of hypercapnia. However, the clinical course, physical examination, brain MRI, whole-body CT, cerebrospinal fluid testing, and hormonal assessment findings helped to effectively rule out numerous diseases. Notably, pseudohyperaldosteronism and diaphragmatic disorders emerged as significant initial findings, suggesting a potential causal relationship. Second, we did not perform direct measurements of diaphragmatic function, such as transdiaphragmatic pressure assessment or ultrasonography, nor did we assess changes in spirometry due to positional changes. Third, we could not completely rule out the potential influence of neuromuscular damage caused by *E. coli* urinary tract infection. Meanwhile, the patient retained her ability to walk and eat, and the predominance of respiratory muscle-related symptoms more strongly implied an association between licorice ingestion and neuromuscular damage.

In conclusion, the study findings suggest that long-term use of licorice in patients with specific underlying issues, including prolonged gastrointestinal transit time, advanced age, and female sex, can lead to severe respiratory failure caused by diaphragmatic dysfunction due to pseudoaldosteronism even if the dosage of licorice is small. These factors should be considered when assessing the potential risks associated with licorice consumption.

## Data Availability

The original contributions presented in the study are included in the article/Supplementary material, further inquiries can be directed to the corresponding author.
